# GDF15 is associated with thyroid cancer progression and may modulate thyroid cancer cell senescence in a p53-dependent manner

**DOI:** 10.3389/fendo.2025.1675245

**Published:** 2025-10-08

**Authors:** Jingyu Ma, Zishuo Liu, Rui Hua, Changyuan Ding, Zhenpeng Yang, Weili Liang, Bin Lv

**Affiliations:** ^1^ Cheeloo College of Medicine, Shandong University, Jinan, Shandong, China; ^2^ Department of Thyroid Surgery, General Surgery, Qilu Hospital of Shandong University, Jinan, Shandong, China

**Keywords:** thyroid cancer, GDF15, cell senescence, p53 pathway, cancer progression

## Abstract

**Background:**

Thyroid cancer, the most prevalent endocrine malignancy, poses significant therapeutic challenges due to its heterogeneous biological behavior. Growth differentiation factor 15 (GDF15), a stress-responsive cytokine, is implicated in tumor progression and senescence regulation in various cancers. However, its role in thyroid cancer, particularly its interaction with the p53 signaling pathway, remains poorly understood. This study aimed to investigate the functional contribution of GDF15 to thyroid cancer progression and its regulatory mechanism in cancer cell senescence.

**Methods:**

Public datasets and clinical specimens were analyzed to evaluate GDF15 expression patterns and their clinical significance. *In vitro* models were established using human thyroid cancer cell lines. GDF15 expression was modulated through siRNA-mediated silencing. RT-qPCR and Western blotting were employed to evaluate the expression levels of target molecules. Functional assays were conducted to assess proliferation (CCK-8, colony formation) and migration/invasion (transwell, cell scratch assay). Cellular senescence was evaluated by measuring β-galactosidase activity, γ-H2AX expression, and senescence-associated secretory phenotype factors. The dependency on p53 was elucidated through siRNA-mediated knockdown of p53. Mechanistic investigations were performed using RNA sequencing and Western blotting.

**Results:**

Compared with adjacent normal tissues, GDF15 was significantly upregulated in thyroid cancer tissues and correlated with lymph node metastasis status. Knockdown of GDF15 suppressed proliferation, migration, and invasion while inducing cellular senescence. RNA sequencing revealed that GDF15 silencing activated the p53 signaling pathway and upregulated p53 expression. Rescue experiments utilizing p53 siRNA partially reversed GDF15-mediated senescence.

**Conclusions:**

GDF15 is implicated in the progression of thyroid cancer and potentially modulates cellular senescence through a p53-dependent mechanism, underscoring its dual functionality as both a pro-tumorigenic driver and a senescence regulator. These findings establish the potential of GDF15 as a therapeutic target and prognostic biomarker in thyroid cancer, providing novel insights developing senescence-centered therapeutic strategies.

## 1 Introduction

Thyroid carcinoma (TC) represents the most frequently diagnosed malignancy within the endocrine system (1). In recent years, the incidence of thyroid cancer has increased rapidly due to advancements in detection and diagnostic technologies, as well as environmental and lifestyle changes (2). Recent studies indicate that thyroid cancer currently ranks third among malignant tumors in Chinese women (3), posing an urgent public health concern. Although most patients with thyroid cancer exhibit favorable prognoses, some still develop extensive lymph node metastasis, extrathyroidal extension, or even distant organ metastasis (4, 5), factors that significantly impair patients’ quality of life and overall survival (6). Therefore, elucidating the molecular mechanisms underlying thyroid cancer progression is critical for identifying diagnostic and therapeutic molecular targets.

Growth differentiation factor 15 (GDF15), a secreted cytokine belonging to the trans-forming growth factor-beta (TGF-β) family, plays pivotal roles in diverse physiological and pathological processes (7, 8). Accumulating evidence highlights its involvement in tumor initiation, progression, and metastasis (9–12). Additionally, GDF15 is closely associated with cellular senescence (13). While genomic studies have suggested GDF15’s potential role in thyroid carcinogenesis and progression, its functional mechanisms have not been fully characterized mechanistically (14, 15).

p53, renowned as the “guardian of the genome,” is a critical tumor suppressor gene in humans. It monitors cellular stress signals—including DNA damage, oncogene activation, and oxidative stress (16, 17)—to regulate downstream target genes, thereby inducing cell cycle arrest, DNA repair, and senescence (18).

Cellular senescence refers to an irreversible cell cycle arrest accompanied by morpho-logical alterations, functional impairments, and secretory profile changes. This state can be induced by diverse intrinsic and extrinsic factors, including telomere shortening, DNA damage, epigenetic dysregulation, and mitochondrial dysfunction. Closely linked to carcinogenesis, senescence acts as a barrier to tumorigenesis but may also facilitate tumor progression under specific contexts (19). Senescent cells exhibit universal hall-marks, including morphological alterations, cell cycle arrest, elevated senescence-associated β-galactosidase (SA-β-Gal) activity, and a senescence-associated secretory phenotype (SASP) (20).

Currently, surgical resection, radioactive iodine (RAI) therapy, and targeted therapy serve as primary strategies for thyroid cancer management. However, their therapeutic efficacy is often limited in advanced cases due to metastasis or drug resistance (4, 6). Recent studies have revealed that cellular senescence reactivation may overcome these therapeutic limitations (21). Previous studies have confirmed that GDF15 promotes thyroid cancer progression through mitochondrial stress and the STAT3 pathway (22). However, whether GDF15 coordinates thyroid carcinogenesis via additional signaling pathways (e.g., p53-dependent mechanisms), particularly its interplay with cellular senescence, remains unexplored. In this study, we observed upregulated GDF15 expression in thyroid cancer tissues compared to normal counterparts. GDF15 levels correlated with cancer cell proliferation, migration, and invasion. Knockdown of GDF15 promoted p53 expression by activating the p53 signaling pathway, thereby triggering cellular senescence. The critical role of the GDF15-p53 axis in senescence regulation identified in this study suggests that targeted inhibition of GDF15 could potentially restore p53-mediated senescence in tumor cells, thereby suppressing tumor progression and improving responsiveness to conventional therapies. Our findings highlight GDF15 as a pivotal contributor to thyroid cancer advancement, positioning it as both a promising diagnostic biomarker and a potential therapeutic target to address the unmet clinical needs in refractory thyroid cancer management.

## 2 Materials and methods

### 2.1 Patients and tumor specimens

A total of 33 thyroid carcinoma tissues and paired adjacent non-cancerous tissues were collected from patients undergoing thyroidectomy at Qilu Hospital of Shandong University between March 2024 and August 2024(**Supplementary Table S1**). Patients with histologically confirmed thyroid carcinoma were included, whereas those presenting a history of concurrent or secondary malignancies were excluded. Written informed consent was obtained from all participants prior to surgical intervention. This study was approved by the Ethics Committee of Qilu Hospital, Shandong University (Approval No. KYLL-202401-041) in accordance with the Declaration of Helsinki principles.

### 2.2 Cell lines and cell culture

Thyroid cancer cell lines TPC-1 was gained from Qilu Hospital. KHM-5M cells were kindly provided by Stem Cell Bank, Chinese Academy of Sciences. The cell lines were authenticated via short tandem repeat (STR) profiling. Mycoplasma testing was conducted monthly. Cells were cryopreserved within 5 passages after subculturing and used within 20 post-thaw passages. Cells were maintained in DMEM (Gibco, Grand Island, NY,USA) supplemented with 10% FBS Gib-co, Grand Island, NY,USA) and 1% penicillin-streptomycin (Gibco, Grand Island, NY,USA), under standard culture conditions at 37 °C with 5% CO_2_ humidified atmosphere.

### 2.3 Bioinformatics analysis of GDF15 expression

The Gene Expression Profiling Interactive Analysis (GEPIA) platform (http://gepia.cancer-pku.cn/) was utilized to examine gene expression patterns, synthesizing RNA sequencing datasets from The Cancer Genome Atlas (TCGA) and Genotype-Tissue Expression (GTEx) initiatives (23). To evaluate differential expression of GDF15 in thyroid cancer, we selected the TCGA-THCA cohort (tumor tissues, n=502) and GTEx thyroid normal tissues (n=337) for comparative analysis. Box plots were generated to visualize expression distribution between tumor and normal groups.

The Tumor Immune Estimation Resource (TIMER) web tool (https://cistrome.shinyapps.io/timer/) was employed to assess immune infiltration levels across cancers (24). In this study, its “Diff Exp” module specifically determined pan-cancer expression patterns of GDF15.

UALCAN database (http://ualcan.path.uab.edu/), a comprehensive portal for cancer transcriptome and clinical data analysis, was utilized to examine GDF15 expression in the “Thyroid carcinoma” dataset through its “Expression Analysis” module (25). Clinicopathological correlations of GDF15 expression were further analyzed based on cancer stage, patient gender, lymph node metastasis status and age subgroups.

### 2.4 Quantitative real-time PCR assay

Total RNA was extracted using the RNAfast 200 Kit (Fastagen, Shanghai, China), followed by cDNA synthesis with HiScript III RT SuperMix for qPCR (+gDNA wiper) (Vazyme, Nanjing, China). Quantitative PCR was performed using ChamQ Universal SYBR qPCR Master Mix (Vazyme, Nanjing, China) on a LightCycler 480 Instrument II (Roche, Switzerland). The thermal cycling protocol consisted of an pre-incubation at 95 °C for 30 sec, followed by amplification in 40 cycles of 10 sec at 95 °C and 30 sec at 60 °C. GAPDH served as the endogenous control for normalization. Relative mRNA expression levels were calculated using the 2^-ΔΔCt^ method. Each sample was run in triplicate. The specific primer sequences are provided in **Table 1**.

**Table 1:** Primers sequences.

Genes	Forward	Reverse
GDF15	CAACCAGAGCTGGGAAGATTCG	CCCGAGAGATACGCAGGTGCA
GADPH	GGAGCGAGATCCCTCCAAAAT	GGCTGTTGTCATACTTCTCATGG
TP53	CCTCAGCATCTTATCCGAGTGG	TGGATGGTGGTACAGTCAGAGC
IL6	AGACAGCCACTCACCTCTTCAG	TTCTGCCAGTGCCTCTTTGCTG
IGFBP3	CGCTACAAAGTTGACTACGAGTC	GTCTTCCATTTCTCTACGGCAGG
CXCL1	AGCTTGCCTCAATCCTGCATCC	TCCTTCAGGAACAGCCACCAGT
CXCL2	GGCAGAAAGCTTGTCTCAACCC	CTCCTTCAGGAACAGCCACCAA
CXCL3	TTCACCTCAAGAACATCCAAAGTG	TTCTTCCCATTCTTGAGTGTGGC

### 2.5 Western blot

The cells or tissues were lysed on ice using RIPA buffer (Beyotime, Shanghai, China) supplemented with the ProteLytic Protease and Phosphatase Inhibitor Cocktail (NCM Biotech, Suzhou, China). Following lysis, cellular debris was removed by centrifugation at 13,000 × g (5 min, 4 °C). The clarified supernatants were mixed with SDS-PAGE loading buffer (20% final concentration) and denatured by boiling at 95 °C for 5 min. Proteins were resolved on 10% Bis-Tris gels (Epizyme, Shanghai, China) using constant voltage (80V) for 2 h through discontinuous electrophoresis. Electrophoretic transfer onto PVDF membranes (0.2 μm pore size; Millipore, USA) was performed using standard wet conditions. After blocking with 5% non-fat milk, membranes underwent sequential incubation with: (1) primary antibodies (overnight at 4 °C) and (2) HRP-conjugated secondary antibodies (1 h at room temperature). Signal detection was achieved using enhanced chemiluminescence ECL reagent(Millipore, Billerica, MA, USA) following manufacturer’s protocols. Primary antibodies included GDF-15 polyclonal antibody (1:20000, cat.no. 27455-1-AP), p53 polyclonal antibody (1:20000, cat.no.10442-1-AP), Lamin B1 Polyclonal antibody (1:50000, cat.no.12987-1-AP) and β-tubulin polyclonal antibody (1:10000, cat.no.10094-1-AP) were purchased from Proteintech (Wuhan, China).

### 2.6 Immunohistochemical (IHC) analysis

Paraffin-embedded tissue sections underwent sequential dewaxing using xylene followed by rehydration through an ethanol gradient. Endogenous peroxidase activity was quenched with 3% hydrogen peroxide. Heat-mediated antigen retrieval was conducted in 10 mM sodium citrate buffer (pH 6.0) for epitope exposure. After blocking non-specific binding sites for 60 min at ambient temperature, primary antibodies were applied and maintained at 4 °C for 16–18 h. Following PBS washes (3 × 5 min), horseradish peroxidase-conjugated secondary antibodies were incubated for 30 min at 25 °C. Chromogenic development was achieved using a commercial DAB detection kit (Servicebio, Wuhan, China), with microscopic evaluation of staining intensity. Quantitative analysis of immunohistochemical results was performed using the Aipathwell digital pathology platform (Servicebio, Wuhan, China).

### 2.7 Cell viability assay

Cell proliferation rates were quantified using the CCK-8 assay (SuperKine™ Maximum Sensitivity Cell Counting Kit-8, Abbkin, China). Cells were initially plated in 96-well culture plates at 2×10³ cells/well and allowed to attach. Following the manufacturer’s guidelines, 10 μL CCK-8 reagent was added per well, and plates were incubated under standard culture conditions (37 °C, 5% CO_2_) for 2h. Cellular metabolic activity was determined by measuring optical density (OD) at 450 nm using a microplate reader.

### 2.8 Cell colony formation assay

Cells in exponential growth phase were plated at 3×10³ cells/well in 6-well culture plates. Cultures were incubated under physiological conditions (37 °C, 5% CO_2_) for 14–20 days until macroscopic colony formation was evident. Colonies were then immobilized with 4% paraformaldehyde (PFA) and visualized through 1% crystal violet staining. The number of cell colonies was counted using Image J software version 8.0.

### 2.9 Small interfering RNA (siRNA) transfection

Cells were seeded in 6-well plates at 2×10^5^ cells per well and cultured overnight. The following day, the medium was replaced with serum-free medium containing siRNA-Lipofectamine 2000 complexes (Thermo Fisher Scientific, Waltham, MA, USA), followed by incubation under standard conditions. The knockdown efficiency of siRNAs was tested by RT-qPCR and western blot analysis. The target siRNA was synthesized by Gemma Genetics (Suzhou, China), with specific sequences provided in **Table 2**.

**Table 2:** The siRNA sequences.

Name	Sense	Antisense
Negative control	UUCUCCGAACGUGUCACGUTT	ACGUGACACGUUCGGAGAATT
siGDF15	AUCCCAUGGUGCUCAUUCATT	UGAAUGAGCACCAUGGGAUTT
sip53	CCACCAUCCACUACAACUATT	UAGUUGUAGUGGAUGGUGGTT

### 2.10 Transwell migration and invasion

Transwell chambers (Corning, NY, USA) were utilized for migration and invasion assays. The upper chamber was filled with serum-free medium, while the lower chamber contained medium supplemented with 10% fetal bovine serum (FBS). For migration assays, 3×10^4^ cells were seeded in the upper chamber. For invasion assays, 1×10^5^ cells were suspended in Ceturegel^®^ Matrix LDEV-Free (Yeasen, Shanghai, China)-coated upper chambers. After 24 h incubation under standard culture conditions, migrated/invaded cells on the membrane underside were fixed with 4% paraformaldehyde, stained with 1% crystal violet for 15 min, and quantified by ImageJ software version 8.0.

### 2.11 Cell scratch assay

Cells were seeded in 6-well plates at 1×10^5^ cells per well and allowed to form a confluent monolayer within 12h. A straight scratch wound was generated across the monolayer using a sterile 200-μL pipette tip. Following this, cells were washed twice with PBS to remove detached cells, and fresh serum-free medium was added. Images of the wound area were captured at predefined time points under an inverted microscope, and migration efficiencies are compared.

### 2.12 RNA-seq analysis

Total RNA was isolated from siGDF15 and negative control cells using TRIzol reagent (Invitrogen, USA). Three biological replicates per group were sent to LC-Bio Technology CO., Ltd (Hangzhou, China) for sequencing library preparation. RNA concentration and purity were assessed using NanoDrop ND-1000 (NanoDrop, Wilmington, DE, USA) and Bioanalyzer 2100 (Agilent, CA, USA). High-quality RNA samples with RNA integrity number (RIN) >7.0 were selected for library construction. Transcriptome sequencing was performed on the Illumina NovaSeq™ 6000 platform (LC-Bio) with paired-end 150 bp (PE150) sequencing mode. The average RNA-seq depth was 42 million reads per sample. Gene assembly and transcript quantification were conducted using StringTie software (https://ccb.jhu.edu/software/hisat2) with fragments per kilobase of transcript per million mapped reads (FPKM) values. Differentially expressed genes (DEGs) were identified using thresholds of |log2(fold change)| ≥1 with statistical significance (p-value <0.05;corrected for multiple testing). Functional annotation of DEGs was performed through Gene Ontology (GO), Kyoto Encyclopedia of Genes and Genomes (KEGG) pathway enrichment analysis, and Gene Set Enrichment Analysis (GSEA). The RNA-seq data was uploaded to the GEO section of the NCBI web server. The GEO accession number was GSE307253.

### 2.13 LinkedOmics analysis

LinkedOmics platform (http://www.linkedomics.org) is a multi-omics resource integrating 32 TCGA-derived cancer datasets (26). The GSEA module within LinkedOmics was employed to identify significantly enriched Kyoto Encyclopedia of Genes and Genomes (KEGG) pathways associated with GDF15 expression in the TCGA-THCA cohort. This approach evaluates coordinated upregulation or downregulation of predefined gene sets, thereby overcoming the limitations of single-gene analyses. KEGG pathway annotations were utilized to map biological interactions and metabolic networks, with pathway visualization highlighting hierarchical relationships. The grade standards were as follows: false discovery rate<0.05; number of simulations: 500.

### 2.14 Protein–protein interaction network and molecular docking

The STRING database(http://string-db.org) was used to construct the protein-protein interaction (PPI) network of GDF15 (27). PPI pairs with an interaction score >0.40 were chosen to be visualized. In the molecular docking section, the corresponding amino acid sequences were retrieved from the RCSB PDB database (http://www.rcsb.org/).

### 2.15 β-Galactosidase staining

Cells were quantified, harvested, and seeded into 6-well plates at a density of 1 × 10^5^ cells per well, followed by incubation under standard culture conditions for 12 hours. After two washes with PBS, adherent cells were fixed with 4% paraformaldehyde at room temperature for 15 min, washed three times with PBS, and stained for SA-β-Gal activity using a commercial kit (C0602, Beyotime) according to the manufacturer’s protocol (28). SA-β-Gal-labeled cells were incubated at 37 °C with the working solution of β galactosidase plus X-Gal for 12 h. After final PBS washes to remove residual stain, senescence-positive cells were visualized and documented using an Axio Scope A1 micro scope (Zeiss, Germany) at a magnification of 20×, with their counts normalized to the total cell number. All experiments were conducted in triplicate.

### 2.16 γ-H2AX immunofluorescence

Cells were seeded into 6-well plates. DNA damage was evaluated using a commercial γ-H2AX immunofluorescence assay kit (C2035S, Beyotime) according to the manufacturer’s protocol (29). Briefly, after fixation with 4% paraformaldehyde, cells were blocked with immunostaining blocking solution at room temperature for 10 min and incubated with anti-γ-H2AX rabbit monoclonal antibody (C2035S-4, Beyotime) overnight at 4 °C. Nuclei were counterstained with DAPI (C2035S-6, Beyotime). A fluorescence microscope (Axio Scope A1 micro scope, Zeiss, Germany) was employed for observation and data recording. All experiments were performed with three replicates.

### 2.17 Statistical analysis

Quantitative data were analyzed using GraphPad Prism v9.0 (GraphPad Software, CA, USA) and expressed as mean ± SD. Group comparisons were assessed by unpaired two-tailed Student’s t-test. For multi-group comparisons, statistical differences were evaluated using one-way ANOVA with Tukey’s *post-hoc* tests. Statistical significance was defined as p < 0.05.

## 3 Results

### 3.1 GDF15 is highly expressed in thyroid cancer and associated with lymph node metastasis

**Figure 1:**
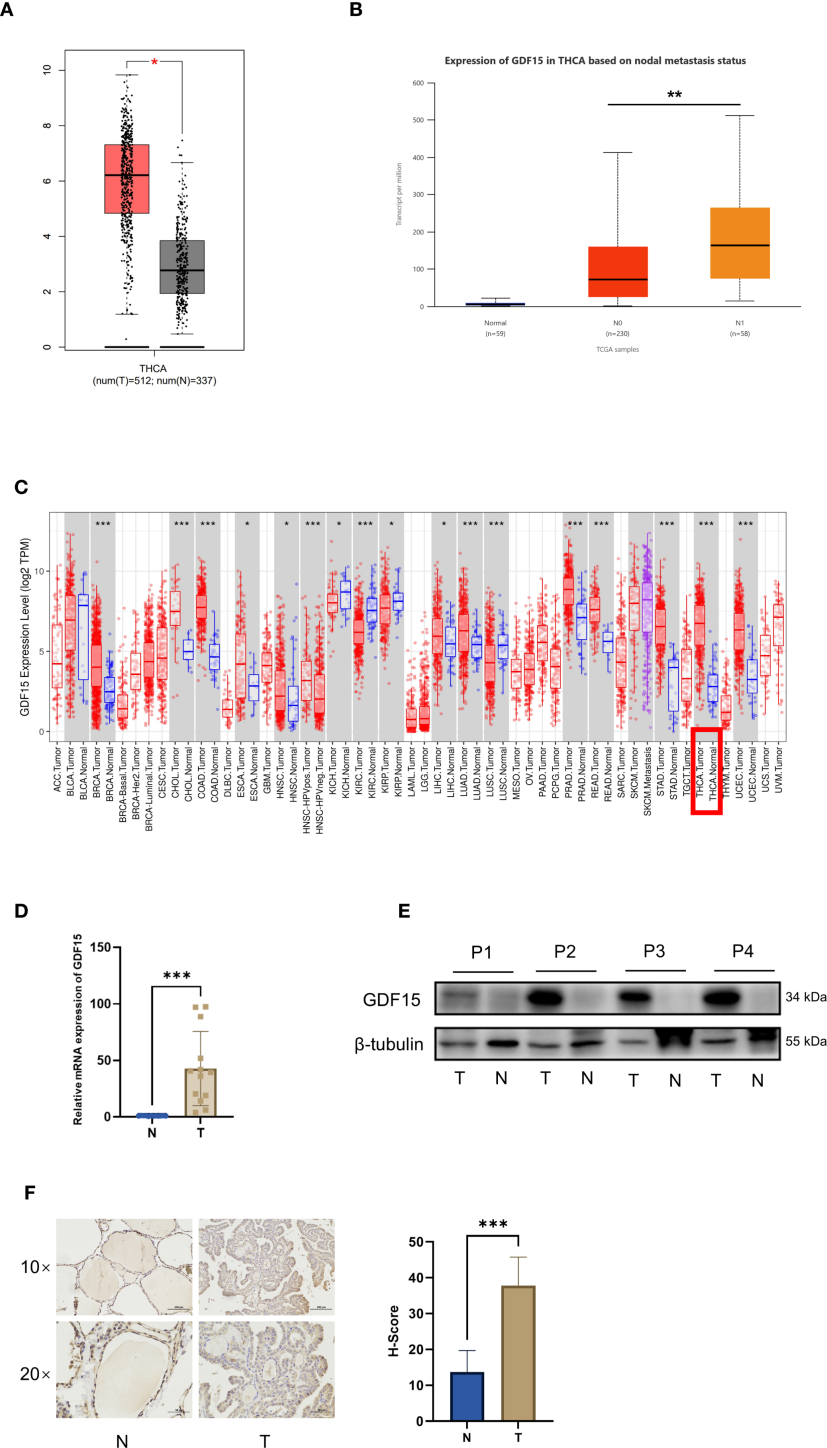
Expression of GDF15 in thyroid cancer and precancerous tissues, and its relationship with clinicopathologic parameters. **(A)** Expression of GDF15 in tumor tissue (red) and adjacent normal tissue (gray), analyzed by GEPIA; **(B)** Expression of GDF15 in TC based on lymph node metastatic status analyzed by UALCAN **(C)** Human GDF15 expression levels in different tumor types from TCGA database were determined by TIMER **(D)** mRNA expression of GDF15 in human thyroid cancer and precancerous tissues (n=12,*p<0.05, **p<0.01, ***p<0.001); **(E)** Determination and quantification of GDF15 protein levels in PTC tissues and paired nontumor tissues by western blotting (T refers to tumor, and N refers to nontumor); **(F)**. Representative immunohistochemistry staining of GDF15 in TC tissues (scale bar, 50 µm, 100 µm) and quantitative analysis of immunohistochemical results (n=6,*p<0.05, **p<0.01, ***p<0.001).

To identify key genes involved in thyroid carcinogenesis and progression, we first performed transcriptome analysis on five pairs of thyroid cancer and adjacent normal tissues. The results revealed significant upregulation of GDF15 in tumor tissues com-pared with normal counterparts (**Supplementary Figure S1A**). Analysis using the GEPIA database confirmed higher GDF15 mRNA expression in thyroid cancer (**Figure 1A**). Further investigation through the TIMER database demonstrated elevated GDF15 expression across multiple cancers, with particularly high levels in thyroid cancer (**Figure 1C**). UALCAN analysis showed no significant correlation between GDF15 expression and patient gender, age, or tumor stage, but identified a strong association with lymph node metastasis status (**Figure 1B**; **Supplementary Figure S1B**).

Consistent with bioinformatics findings, qRT-PCR analysis confirmed markedly higher GDF15 mRNA levels in clinical thyroid cancer specimens than in adjacent normal tis-sues (**Figure 1D**). Western blot and immunohistochemistry (IHC) further validated elevated GDF15 protein expression in tumor tissues (**Figures 1E, F**). Collectively, these results demonstrate that GDF15 is overexpressed in thyroid cancer compared to non-cancerous controls, suggesting its potential involvement in thyroid tumorigenesis and progression.

### 3.2 Knockdown of GDF15 suppresses proliferation, migration, and invasion in TC cells

**Figure 2:**
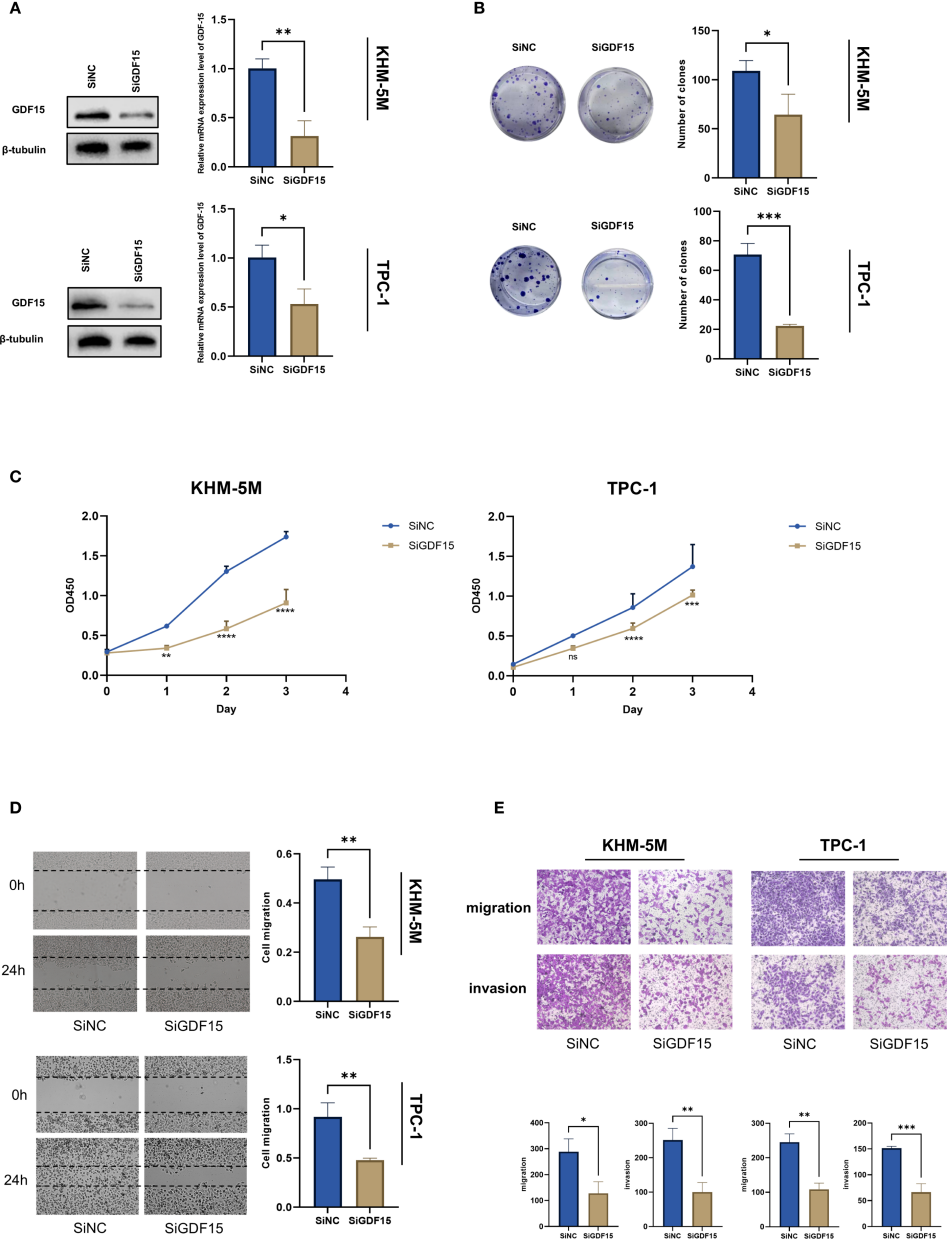
GDF15 knockdown suppresses TC cell proliferation, migration and invasion. **(A)** Western blot analysis of S1PR1 expression in negative control and GDF15 knockdown cells; **(B)** The proliferation capacity of TC cells treated with since or siGDF15 was detected by colony formation assays; **(C)** CCK8 assay was used to detect the proliferation ability of negative control and GDF15 knockdown cells. Counting was performed on days 1, 2, and 3 after inoculation; **(D)** Scratch test analysis of the migration ability of GDF15 knockdown cells; **(E)** Invasion and migration assays were conducted to evaluate the effect of GDF15 knockdown on the metastatic ability of TC cells (magnification 200×). For all studies n=3. Data are presented as means ± SD. Bar chart data were compared by Student’s t-test or ANOVA (ns = not significant, * p < 0.05, ** p < 0.01, and *** p < 0.001, **** p < 0.0001).

To further investigate the functional role of GDF15 in thyroid cancer progression, siRNA-mediated knockdown of GDF15 was performed in thyroid cancer cell lines TPC-1 and KHM-5M. qRT-PCR and Western blot analyses confirmed a significant re-duction in GDF15 expression at both mRNA and protein levels post-knockdown (**Figure 2A**). Colony formation assays revealed that GDF15 silencing markedly suppressed the colony-forming capacity of thyroid cancer cells (**Figure 2B**). CCK-8 cell proliferation assays demonstrated attenuated proliferation in GDF15-deficient cells. To evaluate the impact of GDF15 knockdown on migratory and invasive abilities, wound healing and Transwell assays were conducted. The results demonstrated that GDF15 depletion significantly inhibited the migration and invasion capabilities of thyroid cancer cells (**Figures 2C, D**). These findings collectively demonstrate the critical involvement of GDF15 in regulating proliferation, migration, and invasion in thyroid cancer cells.

### 3.3 Knockdown of GDF15 upregulates p53 expression in TC cells

**Figure 3:**
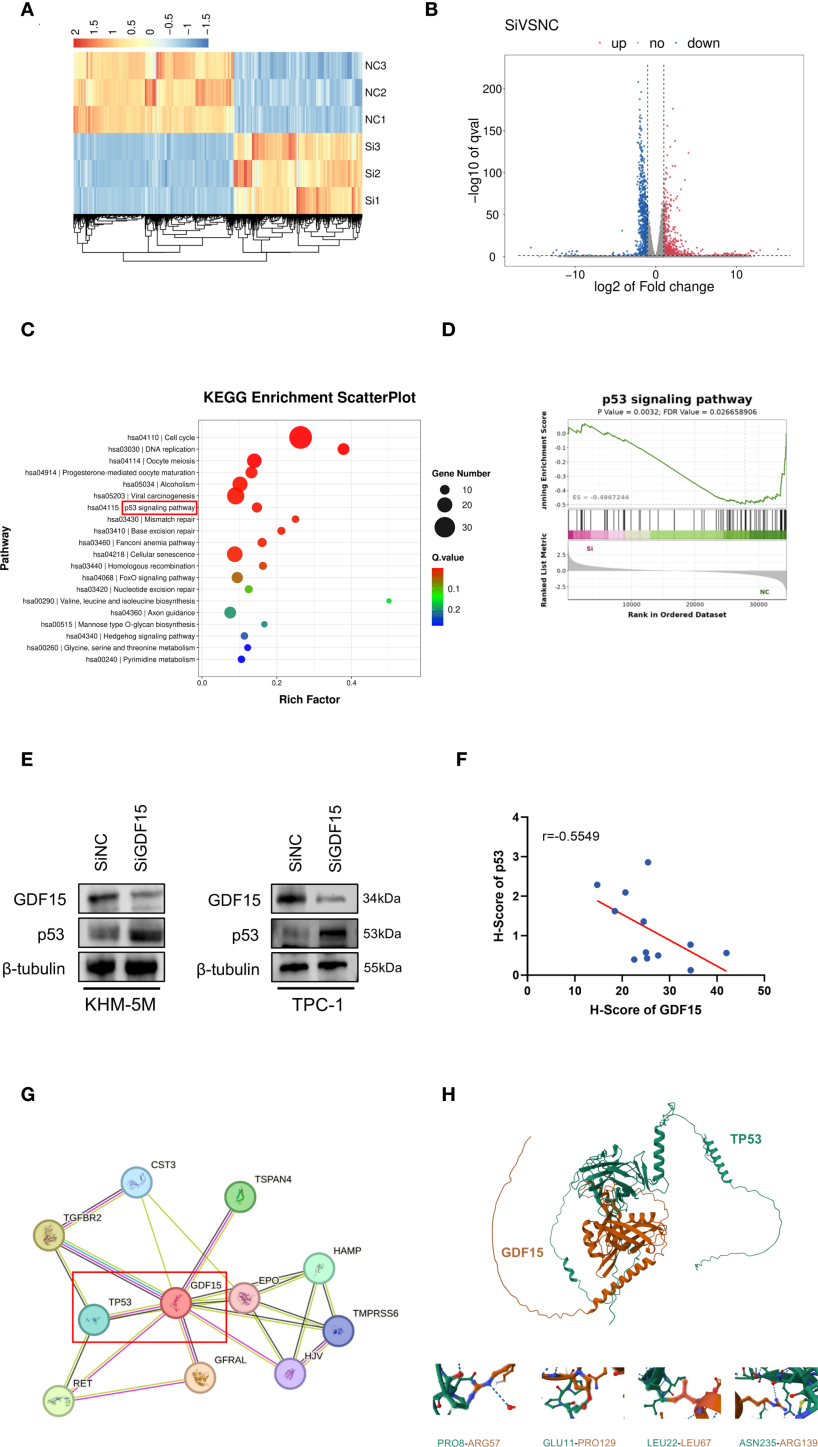
GDF15 knockdown affects the p53 signaling pathway.**(A)** Heatmap analysis of expression differential genes regulated by GDF15 in TPC-1 cells; **(B)** Volcano plot showing 427 upregulated (red) and 757 downregulated (blue) genes; **(C)** KEGG pathway analysis of the DEGs, the top 20 enriched pathways, and KEGG main class; **(D)** GSEA analysis was performed using cells with GDF15 knockdown and vector control cells; **(E)** Western blot demonstrating increased p53 protein levels upon GDF15 knockdown; **(F)** Immunohistochemical analysis of the relationship between GDF15 and p53 expression in thyroid cancer tissues(n=12);**(G)** Protein-protein interaction network of GDF15 predicted by STRING database;**(H)** Mold-predicted TP53-GDF15 heterodimer interface stabilized by key residue pairs.

To further explore the downstream molecular mechanisms by which GDF15 contributes to thyroid cancer progression, we performed transcriptome sequencing analysis of gene expression changes in GDF15-knockdown cell lines. Heatmap visualization revealed substantial alterations in downstream gene expression profiles upon GDF15 silencing compared with controls (**Figure 3A**). Differential expression analysis identified 427 upregulated and 757 downregulated genes in the siGDF15 group versus controls (**Figure 3B**). KEGG pathway analysis of these differentially expressed genes demonstrated significant enrichment in cell cycle regulation, DNA replication, p53 signaling pathway, and cellular senescence-related genes (**Figure 3C**). Consistently, GSEA analysis confirmed upregulation of p53 signaling pathway-associated genes in siGDF15-treated cells (**Figure 3D**). External validation using the LinkedOmics tool for KEGG and GSEA pathway analysis indicated that GDF15 downregulation positively correlated with activation of the p53 signaling pathway (**Supplementary Figure S1C**).

Previous studies have demonstrated that GDF15 is a transcriptional target of p53. Two p53 recognition motifs (RE1 and RE2) are located in its promoter region, with RE2 specifically mediating p53-dependent transcriptional activation (12, 30–32). Multiple re-ports establish that p53 regulates GDF15 expression to influence malignant tumor progression (12, 33, 34). However, whether GDF15 reciprocally modulates p53 remains unexplored. Measurement of p53 mRNA and protein levels in GDF15-knockdown cells revealed increased p53 protein expression without corresponding changes in mRNA abundance (**Figure 3E**; **Supplementary Figure S1D**). Immunohistochemical scoring of clinical thyroid cancer specimens further supported an inverse correlation between GDF15 and p53 expression (**Figure 3F**).

Protein-protein interaction (PPI) network analysis via the STRING database suggested potential physical interactions between GDF15 and p53 (**Figure 3G**). Leveraging known protein structures of GDF15 and p53, DMFold (35) was employed to predict their binding interfaces. In the simulated TP53-GDF15 heterodimer complex, potential docking regions—including PRO8-ARG57, GLU11-PRO1239, LEU22-LEU67, and ASN235-ARG139—were predicted to stabilize the structural integrity of the TP53-GDF15 heterodimer (**Figure 3H**).

Collectively, these findings indicate that GDF15 suppresses p53 protein stability through negative feedback regulation. Its knockdown activates the p53 signaling pathway, triggering cell cycle arrest and senescence. Clinical data and structural modeling further suggest protein-level interactions between GDF15 and p53.

### 3.4 Knockdown of GDF15 induces cellular senescence in TC cells

**Figure 4:**
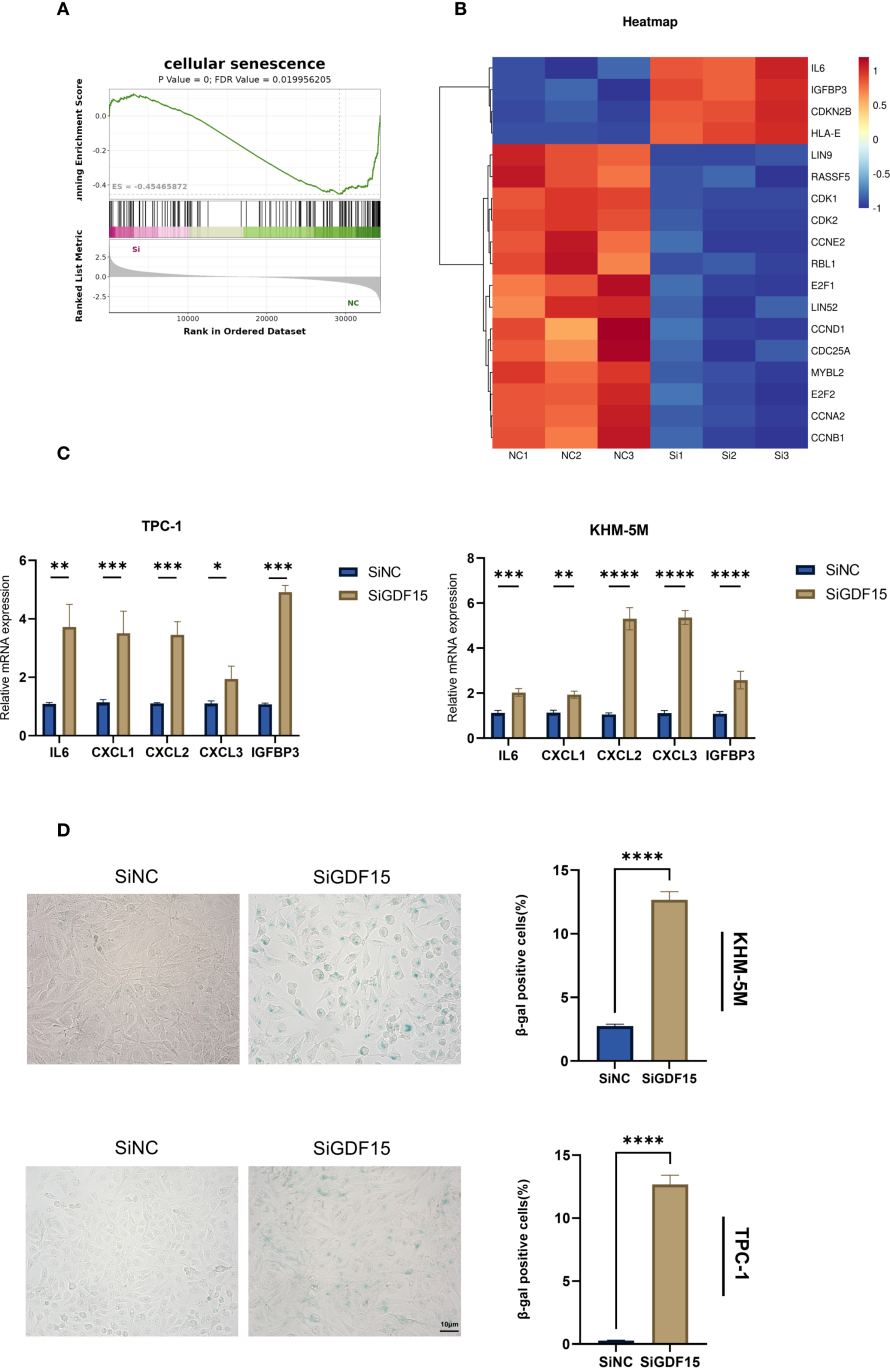
GDF15 is involved in cellular senescence in TC cells. **(A)** GSEA analysis illustrates enrichment of cellular senescence pathways; **(B)** Heatmap analysis of gene expression changes associated with cellular senescence; **(C)** The expression changes of SASP were examined using RT-qPCR; **(D)** β-galactosidase staining was used to qualitatively (left) and quantitatively (right) analyze senescence changes. For all studies n=3. Data are presented as means ± SD. Bar chart data were compared by Student’s t-test or ANOVA (ns = not significant, * p < 0.05, ** p < 0.01, *** p < 0.001, and **** p < 0.0001).

Based on transcriptome sequencing data, we observed significant enrichment of cellular senescence-related pathways in GDF15-knockdown thyroid cancer cells (**Figures 3C**, **4A**). Heatmap analysis demonstrated substantial alterations in senescence-associated gene expression profiles following GDF15 silencing (**Figure 4B**). GDF15 has been recognized as a biomarker of cellular senescence, particularly as a component of the SASP (36, 37). Senescent cells secrete diverse factors—including pro-inflammatory cytokines/chemokines, growth regulators, and angiogenic mediators—to communicate with their microenvironment and influence neighboring cells, a phenomenon collectively termed SASP (38).To investigate whether GDF15 knockdown modulates SASP, we analyzed SASP-related factors and observed increased expression of IL6, IGFBP3, and CXCL1/2/3 in siGDF15-treated cells (**Figure 4C**). Furthermore, β-galactosidase staining assays confirmed elevated activity of senescence-associated β-galactosidase in GDF15-deficient cell lines (**Figure 4D**). These results collectively demonstrate that GDF15 silencing activates the SASP program and upregulates senescence markers (e.g., β-galactosidase activity), thereby inducing significant cellular senescence in thyroid cancer cells.

### 3.5 GDF15 promotes cellular senescence in a p53-dependent manner

**Figure 5:**
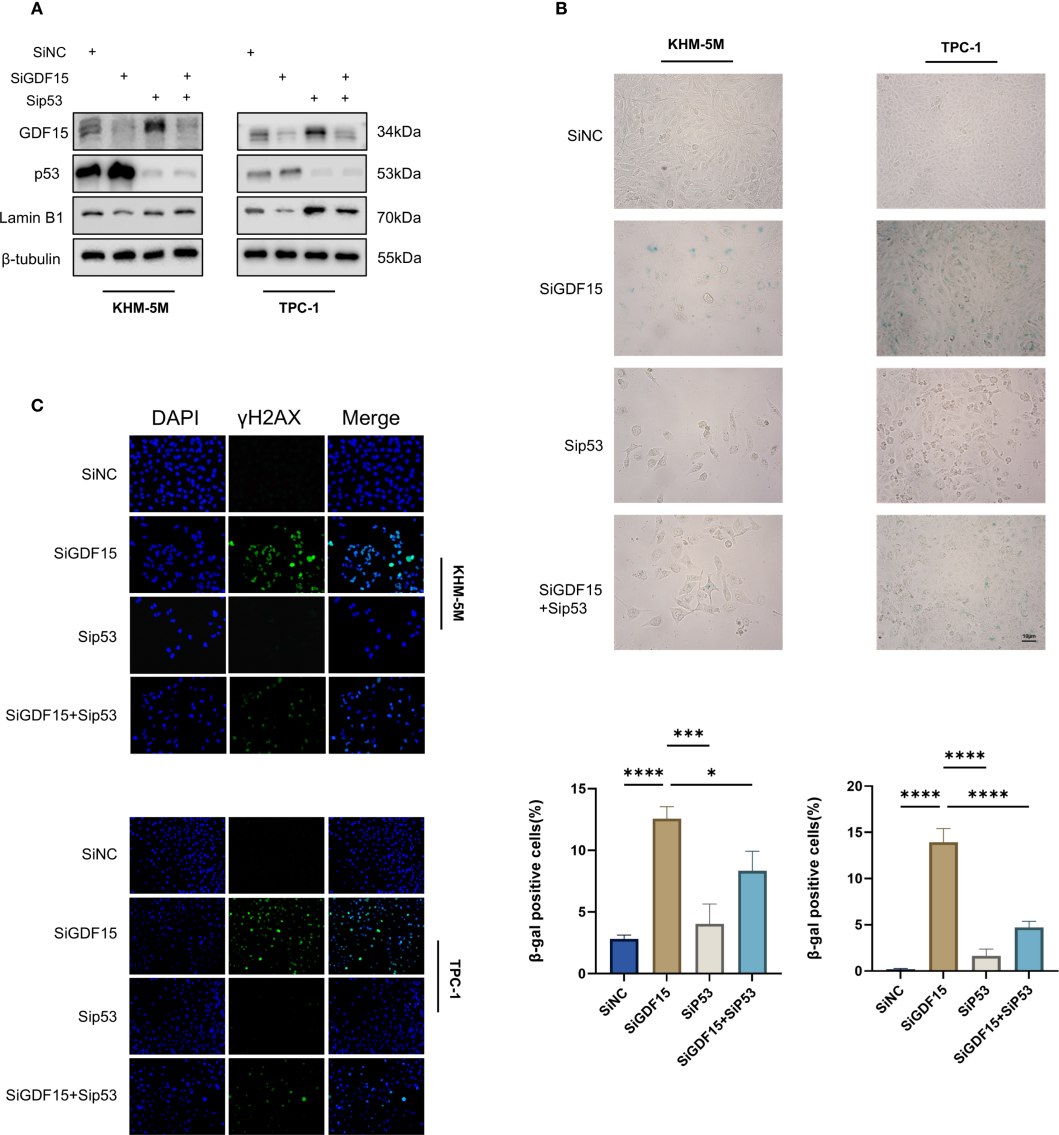
GDF15 regulates TC cell senescence through the p53 pathway. **(A)** The expression levels of GDF15, p53, and LaminB1 were analyzed by Western blot in cells transfected with siGDF15 and sip53; **(B)** Cellular senescence status was evaluated by β-galactosidase staining in cells transfected with siGDF15 and sip53; **(C)** Expression of γ-H2AX was detected using IF staining assay. Representative images of γ-H2AX are shown. Green, γ-H2AX; blue, DAPI. For all studies n=3. Data are presented as means ± SD. Bar chart data were compared by Student’s t-test or ANOVA (ns = not significant, * p < 0.05 and *** p < 0.001).

Given the pivotal role of the p53 signaling pathway in cell cycle arrest and senescence (39), we hypothesized that GDF15 knockdown-induced senescence is p53-dependent. To test this hypothesis, we co-knockdown p53 using siRNA in GDF15-deficient cells. Western blot analysis showed that GDF15 silencing reduced the expression of the senescence marker Lamin B1, whereas concurrent p53 knockdown restored Lamin B1 levels (**Figure 5A**). Consistently, β-galactosidase staining assays revealed that p53 depletion rescued GDF15 knockdown-triggered cellular senescence (**Figure 5B**). Since DNA damage accumulation may drive senescence (40), we further assessed γ-H2AX expression via immunofluorescence. GDF15 knockdown significantly increased γ-H2AX foci formation, which was reversed upon combined p53 silencing (**Figure 5C**). Collectively, these findings demonstrate that GDF15 depletion induces senescence through p53-dependent mechanisms, as evidenced by the reversal of senescence markers (Lamin B1 downregulation, β-galactosidase activity) and DNA damage response (γ-H2AX accumulation) upon p53 co-knockdown. Our results establish a mechanistic link between GDF15-p53 interaction and senescence progression in thyroid cancer cells.

## 4 Discussion

Over the past decade, global thyroid cancer incidence has risen markedly, with Surveillance, Epidemiology, and End Results (SEER) Program data indicating an annual 3% increase in age-standardized incidence rates (41). Although standard therapies—including surgical resection, postoperative TSH suppression therapy, and radioactive iodine ablation—achieve favorable prognoses, refractory local recurrence or distant metastasis persists in subsets of patients with poor outcomes (42).

Prior studies implicate GDF15 in modulating tumor cell behaviors such as proliferation, cell cycle progression, apoptosis, invasion, and metastasis (43–45). Our findings further confirm the critical oncogenic role of GDF15 in the pathogenesis and progression of thyroid cancer (22) and provide expanded mechanistic insight: connecting GDF15 to p53-mediated cellular senescence, offering novel insights into therapeutic strategies targeting this cytokine. The association between elevated GDF15 expression and lymph node metastasis aligns with its established roles in promoting epithelial-mesenchymal transition (EMT) and extracellular matrix remodeling in other malignancies (22, 46, 47). Emerging evidence indicates that GDF exhibits tumor-suppressive properties (12, 45), while its contradictory roles across malignancies underscore its context-dependent functions within distinct tumor microenvironments, thus emphasizing the necessity for developing GDF15-targeted therapeutic strategies in precision oncology. Notably, GDF15 knockout significantly suppressed thyroid cancer cell proliferation, migration, and invasion, indicating its functional involvement in maintaining malignant phenotypes.

This study extends these observations by revealing a unique regulatory axis between GDF15 and tumor suppressor p53 in thyroid cancer. The discovery that GDF15 depletion upregulates p53 expression while inducing senescence unveils a paradoxical relationship between an oncogenic cytokine and a classical tumor suppressor. Mounting evidence suggests p53 reactivation triggers senescence bypass in cancer cells via transcriptional activation of p21 and other senescence-associated secretory phenotype (SASP) factors (39). Our data showing increased SA-β-galactosidase activity and senescence marker alterations in GDF15-knockdown cells support this paradigm. Crucially, the dependency of GDF15-mediated senescence on p53 accumulation highlights a previously unrecognized regulatory mechanism. We hypothesize that GDF15 may suppress p53 through post-translational modifications or transcriptional repression, potentially via MDM2-mediated ubiquitination pathways (48). This hypothesis warrants validation through proteasome inhibition assays and ubiquitination profiling.

Therapeutically, GDF15 inhibition-induced senescence presents dual clinical implications. While senescence may restrict tumor progression by establishing stable growth arrest, emerging evidence cautions that senescent cells paradoxically promote tumor recurrence through SASP-mediated microenvironment remodeling (49). Combining GDF15-targeted therapies with agents neutralizing SASP effects could optimize outcomes—a strategy under investigation in solid tumors (50). Investigating the co-expression patterns and interaction mechanisms between GDF15 and other tumor-regulatory genes holds substantial scientific value in the context of thyroid cancer gene expression characteristics (14, 15). Critically, given the central role of BRAF V600E mutations and RET variants in thyroid cancer molecular diagnostics and targeted therapy (51), mechanistic studies exploring functional interactions between GDF15 and these drivers (52, 53) may yield novel biomarkers, prognostic tools, and combination therapeutic targets.

Several limitations warrant acknowledgment. First, our *in vitro* models incompletely replicate the tumor microenvironment influencing GDF15 signaling; more sophisticated *in vivo* systems are required. Second, the clinical correlation between GDF15 expression and patient survival needs validation in larger multicenter cohorts. Third, the precise molecular mechanism linking GDF15 to p53 regulation—whether through direct protein interaction, epigenetic modulation, or intermediate signaling—remains unresolved. Future studies employing chromatin immunoprecipitation (ChIP) and co-immunoprecipitation (Co-IP) assays could clarify these relationships.

In summary, this study elucidates GDF15’s critical role in thyroid cancer progression. Experimental evidence demonstrates significant GDF15 overexpression in thyroid tumors, positively correlating with lymph node metastasis and other clinicopathological features. Functional assays confirm that GDF15 knockdown suppresses proliferation, migration, and invasion while inducing senescence. Mechanistically, GDF15’s oncogenic activity tightly associates with p53 signaling—its depletion upregulates p53 protein levels, and sustained p53 accumulation is prerequisite for senescence induction. Future studies should focus on developing GDF15-specific inhibitors and validating the *in vivo* efficacy, while exploring their synergistic anti-tumor effects with existing therapeutic regimens such as tyrosine kinase inhibitors (TKIs). This may provide novel avenues for advanced thyroid cancer treatment. Furthermore, the broad implications of GDF15-mediated senescence suppression in cancer progression and treatment resistance require further investigation. Exploring these directions may accelerate the clinical translation of senescence-targeted strategies.

## Data Availability

The datasets presented in this study can be found in online repositories. The names of the repository/repositories and accession number(s) can be found in the article/**Supplementary Material**.
