# Supplementary material for: GDF15 is associated with thyroid cancer progression and may modulate thyroid cancer cell senescence in a p53-dependent manner
Source: Front Endocrinol (Lausanne). 2025 Oct 8;16:1675245. doi: 10.3389/fendo.2025.1675245 (PMC12540127; doi:10.3389/fendo.2025.1675245)
Supplement: Supplementary file 1 [file DataSheet1.docx]

Supplementary Material

# Supplementary Figures and Tables


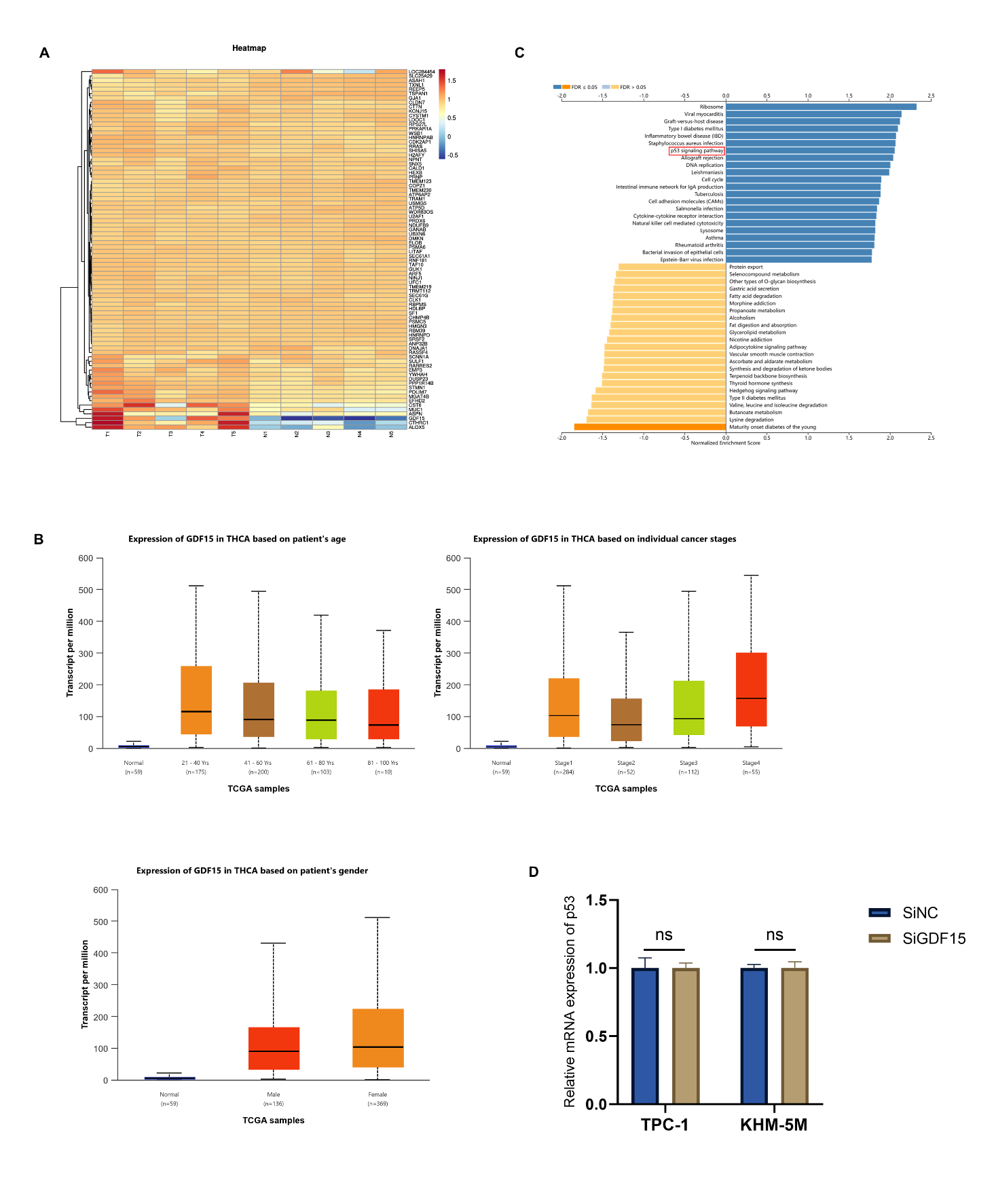


**Supplementary Figure 1.** (A) Heatmap showing expression of representative genes in thyroid cancer and paraneoplastic tissues; (B) Expression of GDF15 in TC based on patient’s age, gender and cancer stages analyzed by UALCAN; (C) LinkedOmics was used to analyze the KEGG pathways of GDF15 in THCA; (D) Changes in p53 mRNA levels following GDF15 knockdown were assessed by qRT-PCR.

**Supplementary Table 1.** Clinicopathological features of patients.

| Clinicopathological features | Number of cases (n=33) |
| --- | --- |
| Age |  |
| <55 | 26 (78.8%) |
| ≥55 | 7 (21.2%) |
| Gender |  |
| Male | 6 (18.2%) |
| Female | 27 (81.8%) |
| T stage |  |
| T1a | 26 (78.8%) |
| T1b | 4 (12.1%) |
| T2 | 3 (9.1%) |
| N stage |  |
| N0 | 18 (54.5%) |
| N1a | 12 (36.4%) |
| N1b | 3 (9.1%) |
| M stage |  |
| M0 | 33 (100.0%) |
| TNM stage |  |
| Ⅰ | 31 (93.9%) |
| Ⅱ | 2 (6.1%) |
